# A case report of intestinal obstruction caused by cryptogenic multifocal ulcerous stenosing enteritis

**DOI:** 10.1186/s12876-020-01450-5

**Published:** 2020-09-15

**Authors:** Cheng Chang, Chen Jiang, Yaoyao Miao, Bin Fang, Lili Zhang

**Affiliations:** 1 General Surgery, Qingdao Municipal Hospital (Group), Qingdao, 266011 Shandong Province China; 2 Pathology Department, Qingdao Municipal Hospital (Group), Qingdao, 266011 Shandong Province China; 3 Infectious Diseases Department, The Affiliated Qingdao Hiser hospital of Qingdao University (Qingdao Hospital of Traditional Chinese Medicine ), Qingdao, 266033 Shandong Province China; 4 Department Of Anus & Intestine Surgery, The Affiliated Qingdao Hiser hospital of Qingdao University, (Qingdao Hospital of Traditional Chinese Medicine ), Qingdao, 266033 Shandong Province China; 5 Department of Radiology, The Affiliated Qingdao Hiser hospital of Qingdao University (Qingdao Hospital of Traditional Chinese Medicine ), Renmin Road 4, Qingdao, 266033 Shandong Province China

**Keywords:** Cryptogenic multifocal ulcer stenosing enteritis, Intestinal obstruction, Small intestinal ulcer, Small intestinal stenosis

## Abstract

**Background:**

Cryptogenic multifocal ulcer stenosing enteritis (CMUSE) is a rare disease characterized by multiple superficial ulcers, stenosis, and obstruction of the small intestine of unknown origin, and the course can recur.

**Case presentation:**

We encountered a 62-year-old male patient with intestinal obstruction. The patient was admitted to the hospital for surgical treatment due to intestinal obstruction, and was diagnosed with cryptogenic multifocal ulcer stenosis enteritis due to comprehensive surgery and postoperative pathological considerations.

**Conclusion:**

In the future, we will continue to follow up the patient. The present study aims to remind clinicians of this disease, and reduce the incidence of misdiagnosis.

## Background

Cryptogenic multifocal ulcerous stenosing enteritis (CMUSE) is a rare idiopathic disease, At present, there are only over 60 cases of CMUSE reported in the world [1], this was first reported in 1964 [2]. This is featured by unexplained small bowel multiple superficial ulcers, stenosis, obstruction, and no biological signs of systematic inflammation, which are prone to recurrence. Hormone therapy is effective for some patients [3]. We encountered a patient with intestinal obstruction, which was very severe, and this patient was admitted to the hospital for immediate surgical treatment. Combined with the intraoperative findings and postoperative pathological diagnosis, the patient was diagnosed as CMUSE.

## Case presentation

**Fig. 1:**
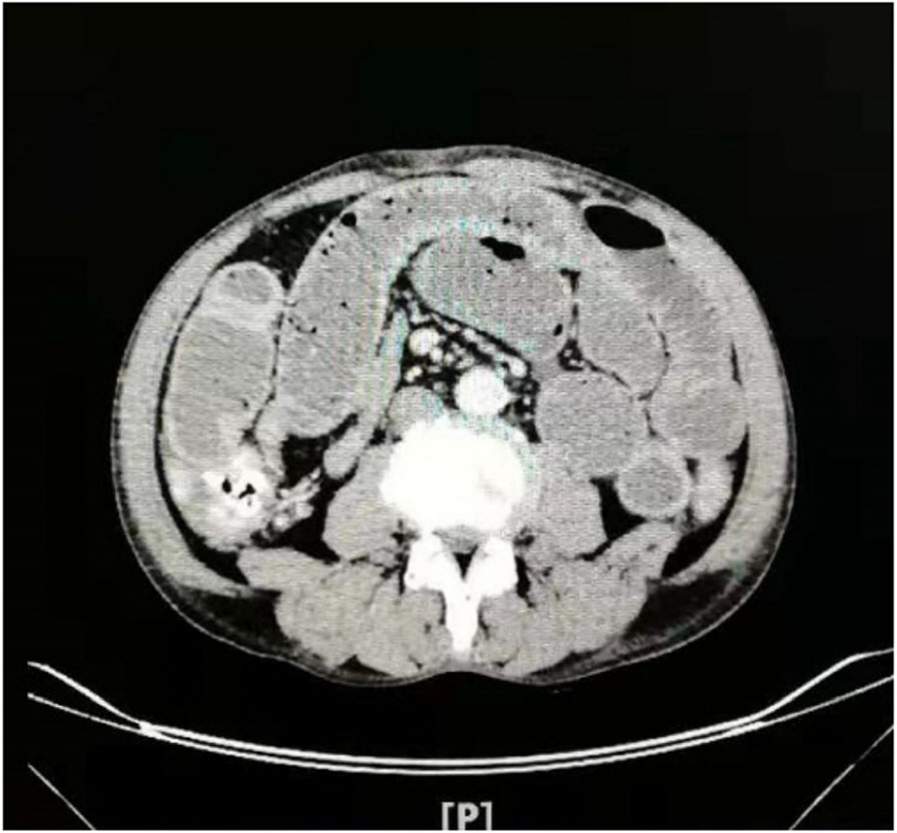
Preoperative abdominal CT examination showed small bowel obstruction

A 62-year-old male patient was admitted to the hospital due to “abdominal pain and abdominal distension for one month, with aggravation for five days”. The patient presented with abdominal pain in the previous one month without obvious inducement, mainly around the umbilicus, paroxysmal, accompanied by abdominal distension, with a small amount of exhaust and defecation. The patient continued to eat without paying attention to this. Five days ago, these symptoms aggravated, and the patient stopped venting and defecating. The patient visited our hospital for treatment and underwent abdominal CT, which revealed small intestinal obstruction (Fig. 1). Physical examination: abdominal distention, intestinal type, total abdominal tenderness, obvious periumbilical, and bowel sounds hyperactive. Laboratory examination: WBC count: 19.61× 10^9^ /L, hemoglobin: 91 g/L, serum albumin 22 g/L, and the remaining assay examination was normal. Previous history: good health, no history of abdominal surgery, and no history of medication. Since the obstruction was long and severe, emergency laparotomy was performed.

**Fig. 2:**
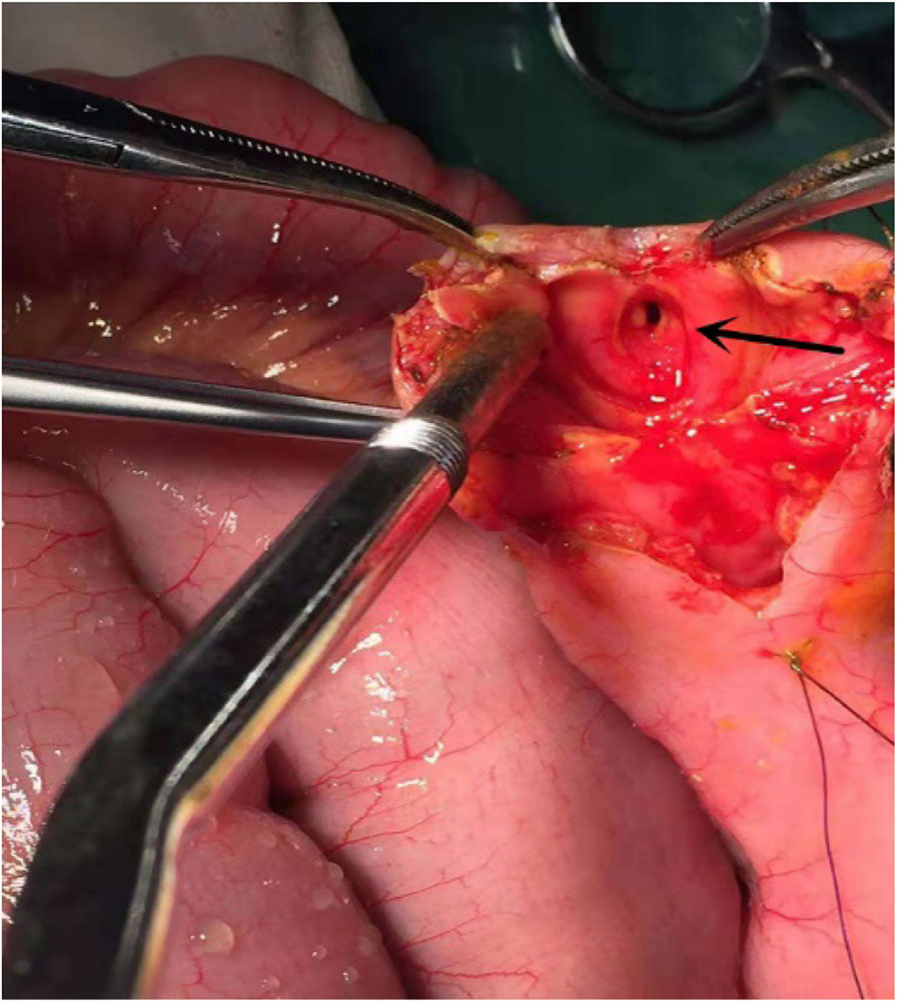
Intraoperative photograph. The black arrow shows “ stenosis ring “

**Fig. 3:**
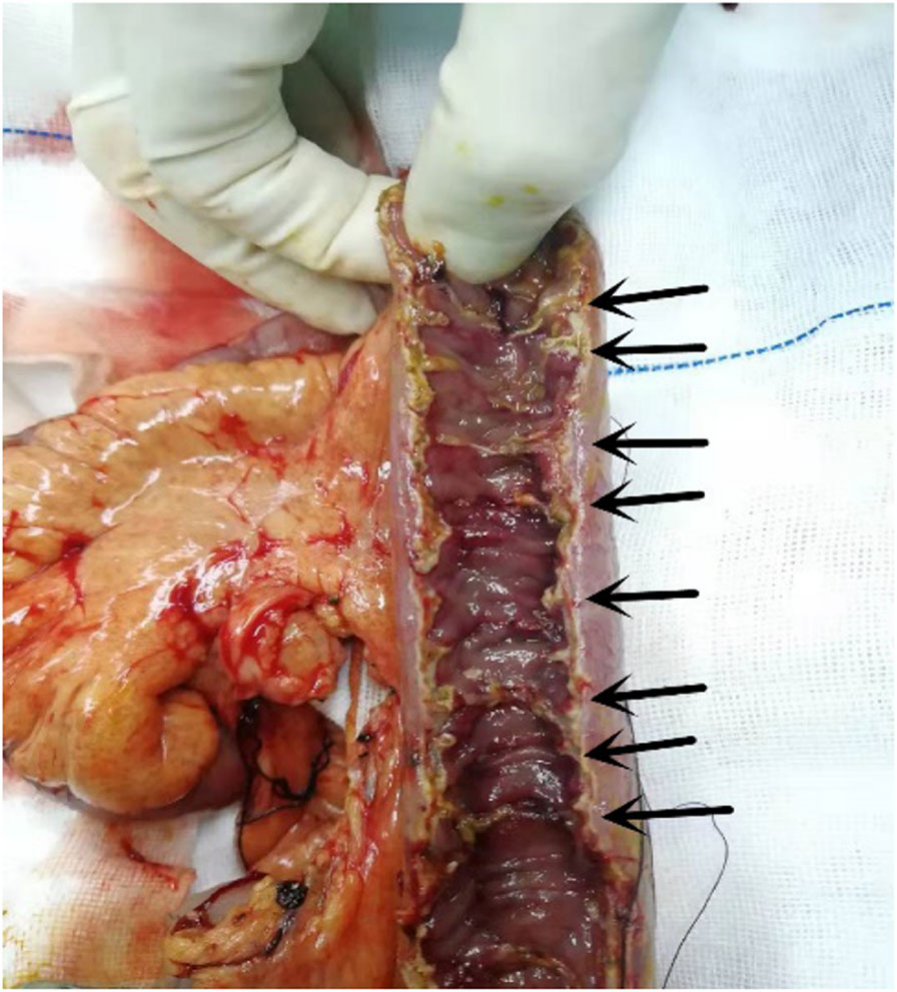
Intraoperative photograph. The black arrow shows “ stenosis ring “(Has been opened)

Observations during the operation: there was no adhesion in the abdominal cavity, there was no twist and compression of the intestine, no tumor was detected, and the obstruction was located in the small intestine at 70 cm away from the ileocecal area. The small intestine above the obstruction was highly dilated, and the diameter of the intestine was 8–10 cm. The appearance of the following small intestine was normal. The intestinal canal was longitudinally dissected at the obstruction, a small amount of food fiber was expelled from the intestinal canal at the obstruction site, and a “stenosis ring” was formed in the small intestinal cavity. Merely pores with a diameter of 0.3–0.4 cm were left, and no obvious abnormality was observed in the intestinal mucosa (Fig. 2). The distal intestine was longitudinally opened to the ileocecal region, and multiple “stenosis rings” were found in the intestinal lumen at approximately 40 cm from the distal obstruction, presenting with the same shape (Fig. 3). The shortest spacing between the “stenosis ring” was 1.0 cm, and the longest spacing was 6 cm. The diseased bowel was removed, and the patient recovered well without discomfort, such as diarrhea. The patient was followed up for two months, and no discomfort was found.

**Fig. 4:**
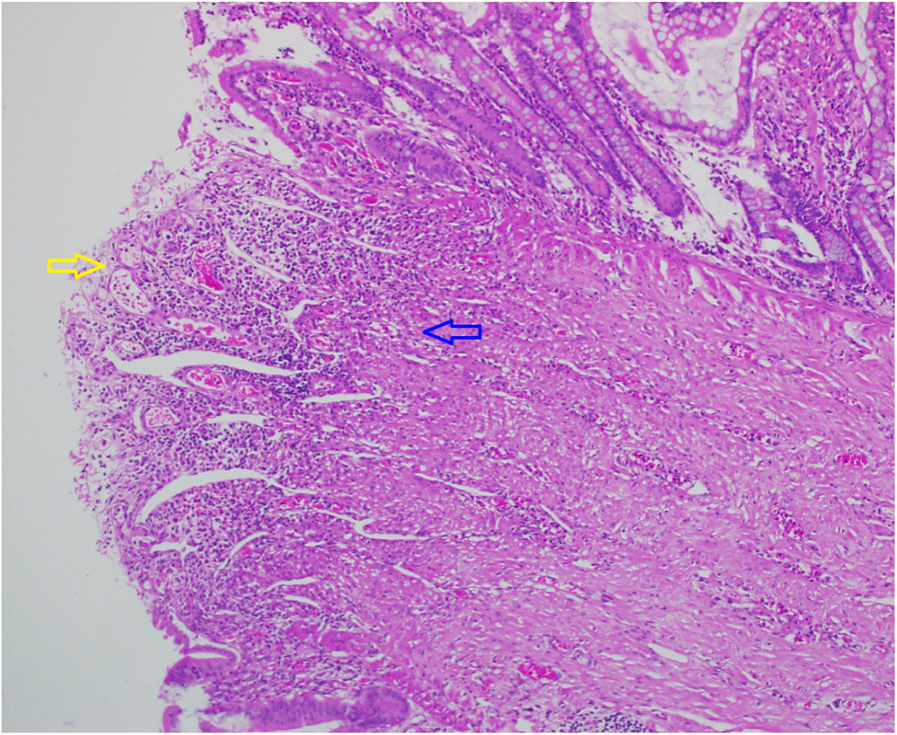
Postoperative pathological photos. HE stain, 100 times magnification. Yellow arrow: shallow intestinal mucosa ulcer. Blue arrow: muscle fibers growing longitudinally below the ulcer.

Pathological findings included: intestinal stenosis was obtained, shallow intestinal segmental ulcer formation, mucosal erosion and inflammatory granulation tissue formation, the mucosal muscle layer disappeared, submucosal fiber hyperplasia, and longitudinal extension to the mucosal layer, causing mucosal uplift and intestinal wall stenosis A large amount of neutrophil infiltration, and vasodilatation and congestion were observed in the serosa, but no obvious structural abnormalities were found in the muscle layer and serosa layer (Fig. 4).

## Discussion and conclusion

The etiology and pathogenesis of CMUSE remains unknown. Some scholars speculate that autoimmune abnormalities, excessive formation of fibrous tissue, vasculitis, and mutations in the gene PLA2G4A encoding cytosolic phospholipase A2-a are correlated to its pathogenesis [3, 4].

Yu Zhang et al. [5] summarized the diagnostic criteria for CMUSE: (1) Medical history: long course; persistent and occult blood loss from the gastrointestinal tract with severe anemia. (2) Clinical features: chronic and relapsing subileus episodes; chronic iron-deficiency anemia with fatigue, edema, or growth retardation, rarely with diarrhea; with normal inflammatory makers or other biological signs of systemic inflammation; normal colon and stomach; no extrointestinal manifestations. (3) Imaging manifestation: In enteroclysis: multiple persisting stenoses of the small intestine caused by brous strictures. (4) Endoscopic findings: multiple, pleiomorphous and superficial ulcers, and sharply demarcated from the surrounding normal mucosa; caution was taken during the capsule endoscopy examination, because this may become stranded in the strictures. (5) Histological findings: necrotic inflammatory ulcers not reaching the proper muscular layer; nonspecific inflammation, and erosion with submucosal fibrosis.

Differential diagnosis could include: (1) Crohn’s disease (CD): CD is one of the most important differential diagnoses for CMUSE. The difference is that Crohn’s disease is transmural inflammation, and the typical lesions are longitudinal and fissured ulcers, which are prone to fistula formation and intestinal perforation. Giant cell granulomas are observed in its pathology [6]. (2) NSAID-associated bowel disease: This disease was very similar to CMUSE under endoscopy, with superficial ulcers and intestinal stenosis [7], but NSAID-associated bowel disease has a long history of medication, and can be recovered after withdrawal [8]. (3) Intestinal tuberculosis can occur with intestinal ulcers or stenosis. Its characteristics include the following: circular ulcers, which can be accompanied by extraintestinal tuberculosis; the histopathological findings of caseous necrotizing granulomatous or positive acid staining could be confirmed. (4) Vasculitis involves multiple ulcers in the intestine, such as Behcet’s disease, granulomatous vasculitis, and systemic scleroderma [9]. However, these ulcers are deep, and may have perforations. Furthermore, multifocal stenosis is rare, and blood vessel inflammation is a systemic disease that often involves multiple systems.

Main treatment methods includes: (1) Hormones: Glucocorticoids are presently the first line of treatment, but these are likely to cause hormone dependence, relapse, and steroid resistance [7]. (2) Endoscopic balloon dilatation: This can alleviate the symptoms, and prevent small bowel resection [3, 4]. (3) Surgery: This can easily relapse after surgery, and repeated surgery can easily cause complications, such as short bowel syndrome [3]. (4) Nutritional support therapy: Enteral or parenteral nutrition and iron supplementation can improve the symptoms in the short term, and even obtain mucosal healing,. However, since the patient resumed eating, the mucosal ulcer, anemia and hypoproteinemia rapidly relapsed [10].

CMUSE is a rare disease of unknown etiology. Multiple superficial ulcers and intestinal stenosis in the small intestine are its typical manifestations. Its diagnosis depends on the comprehensive judgment of clinical manifestations, imaging examinations, endoscopy and pathological examinations. The investigators will continue to follow the patient and monitor for its recurrence. The present study aims to remind clinicians of this disease. When encountering similar lesions, the possibility of this disease must be considered. This also provides a differential diagnosis for small bowel ulcers and stenotic diseases, reducing the occurrence of misdiagnosis.

## Data Availability

This case report contains clinical data obtained from the electronic medical record in Qingdao Municipal Hospital (Group). Additional information is available upon request, but only in accordance with the privacy restrictions of the hospital.

## Abbreviations

CMUSE: Cryptogenic multifocal ulcer stenosing enteritis
CT: Computer tomography
WBC: White blood cell count
cm: Centimeters
CD: Crohn’s disease
NSAIDs: Non-Steroidal Anti-inflammatory Drugs
